# Supplementary material for: Fulminant Adenoviral-Induced Hepatitis in Immunosuppressed Patients
Source: Viruses. 2022 Jul 1;14(7):1459. doi: 10.3390/v14071459 (PMC9323657; doi:10.3390/v14071459)
Supplement: Supplementary file 1 [file viruses-14-01459-s001.zip › viruses-1739408-supplementary.pdf]

Table S1. Primer and probe (TaqMan) for HAdV real-time PCR.

**Primer**
F1 5′ CGA GGA TGC GAG CCG AT 3′
F2 5′ TGC GAG GAT GAG AGC CGA T 3′
R1 5′ ACT TCT ACT TTC ACC ACA TCA ACA GC 3′
R2 5′ ACT TCT ACT TTC ACC ACA TCA ATA GCC 3′
R3 5′ ACT TCT ACT TCC ATC ACA TCA ACA GC 3′
R4 5′ ACT TCT ACT TTC ATC ACA TCA ACA GCC 3′
**Probe**
5′ TGG TGG C(AG)G GA(AG) ATC CAG TTC TTC C 3′

Abbreviations: HAdV – Human Adenovirus; PCR – Polymerase chain reaction.
